# GHOST—Gate to Hybrid Optimization of Structural Topologies

**DOI:** 10.3390/ma12071152

**Published:** 2019-04-09

**Authors:** Bogdan Bochenek, Katarzyna Tajs-Zielińska

**Affiliations:** Institute of Applied Mechanics, Cracow University of Technology, 31-864 Krakow, Poland; Bogdan.Bochenek@pk.edu.pl

**Keywords:** topology optimization, hybrid algorithm, compliance minimization

## Abstract

Although well-recognized in the fields of structural and material design and widely present in engineering literature, topology optimization still arouses a high interest within research communities. Moreover, it is observed that the development of innovative, efficient and versatile methods is one of the most important issues stimulating progress within the topology optimization area. Following this activity, in the present study, a concept of a hybrid algorithm developed in order to generate optimal structural topologies of minimal compliance is presented. The hybrid algorithm is built based on two existing approaches. The first one makes use of the formal optimality criterion, whereas the second one utilizes a special heuristic rule of design variables updating. The main idea that stands behind the concept of the present proposal is to take the advantage of both algorithms capabilities. In a numerical implementation of the hybrid algorithm, the design variables are updated at each iteration step using both approaches, and the solution with a lower objective function value is selected for the next iteration. The numerical tests of the generation of minimal compliance structures have been performed for chosen structures including a real engineering one. It has been confirmed that the proposed hybrid technique based on switching between the considered rules allows the final structures having lower values of compliance as compared with the results of an application of basic algorithms running separately to be obtained. Moreover, based on promising results of the tests performed, one can consider the proposed concept of a hybrid algorithm as an alternative for other existing topology generators.

## 1. Introduction

Topology optimization is a dynamically developing research area with numerous applications to many research and engineering fields ranging from the aeronautical industry, e.g., Reference [1], through civil engineering, e.g., Reference [2], and architecture, e.g., Reference [3], to material science, e.g., Reference [4]. Since the pioneering papers by Bendsoe and Kikuchi [5] and Bendsoe [6] one can find in the literature numerous approaches to generating optimal topologies based both on the optimality criteria and evolutionary methods. A general overview as well as a broad discussion on topology optimization concepts are provided by many survey papers, e.g., References [7,8,9,10]. At the same time numerous papers present a variety of solutions including classic Michell examples as well as complicated spatial engineering structures. Moreover, it is worth observing that topology optimization tools are nowadays present in commercial software like Ansys, Altair-OptiStruct or Abaqus-Atom. Nevertheless, although remarkable achievements have been already made, there is still space for further investigations.

The implementation of efficient and versatile methods to the generation of optimal topologies for engineering structural elements is probably the most important issue stimulating the progress within the topology optimization area. One can observe over the last two decades the implementation of various specific methods ranging from gradient-based approaches, e.g., Reference [6], where mathematical models are derived to calculate the sensitivities of design variables to non-gradient-based ones, where the material is redistributed using various, usually heuristic techniques. In what follows, the generation of optimal topologies involves, among others, evolutionary ESO/BESO structural optimization [11,12], genetic algorithms [13,14], other biologically inspired algorithms [15,16], the material cloud method [17], spline based topology optimization [18], level set method [19,20], cellular automata [21,22,23,24,25,26] or the moving morphable components approach [27,28]. It can be seen from the above that traditional gradient-based mathematical programming algorithms are more readily replaced by novel and efficient heuristic methods inspired by biological, chemical or physical phenomena. Heuristic optimization techniques are gaining popularity among researchers, see, for example, Reference [29], because they are easy for numerical implementation, do not require gradient information and one can easily combine this type of algorithm with any finite element structural analysis code. This practical aspect of engineering the implementation of topology optimization techniques seems to be one of the most important issues for contemporary design.

In the present study, the concept of a hybrid algorithm to generate optimal structural topologies of minimal compliance is proposed. The main idea that stands behind the concept of the present proposal is to take two existing algorithms and to build a new one which takes the advantages of both algorithms capabilities, which finally allows for an improvement of the efficiency of optimal topology generation processes. For the purpose of the present paper, the ones presented by Sigmund in Reference [30] and quite recently proposed by Bochenek and Mazur in Reference [31] have been selected. The first one is based on the formal optimality criterion, whereas the second one utilizes a special heuristic rule of design variables updating. In the numerical implementation of the hybrid algorithm, the design variables are updated at each iteration step using both approaches and the better one from obtained solutions, in terms of objective function value, is selected for the next iteration. With a view to illustrate the concept proposed in this paper, the generation of minimal compliance structures have been performed for selected test structures. Moreover, the application of the hybrid algorithm to the optimization of the exemplary engineering structure has been presented. For this purpose, the algorithm has been combined with the structural analysis code Ansys. It has been confirmed that the proposed hybrid technique based on switching between the considered rules allows final structures having lower values of compliance as compared with the results of the application of basic algorithms running separately to be obtained.

## 2. Problem

The principle of topology optimization is to find a distribution of materials within a design domain which is optimal in some sense. During the optimization process, the material is redistributed from the parts where it is not necessary, from the objective point of view, to the parts where a structural stiffness is required to transfer applied loads to the supports. The topology optimization procedure leads finally to the material/void distribution which can be visualized by black and white regions over the design domain. In topology optimization, the design domain is usually discretized by finite elements, for which design variables $d_{n}$, n=1,2,…,N are selected. When the most common and a very efficient approach called SIMP (Solid Isotropic Material with Penalization, e.g., Reference [7]) is implemented, the design variables are defined as the relative densities of the material. In what follows, as a material representation, the elastic modulus $E_{n}$ of each finite element can be modeled as a function of the relative density $d_{n}$ using the power law:

(1) $$E_{n}=d_{n}^{p}E_{0}$$

The power *p* in Equation (1) penalizes the intermediate densities and drives the design to a material/void structure. The majority of topology optimization results reported in the literature regard structures for which the distribution of material has been generated so as to minimize their compliance *c* under applied loads:

(2) $$\mathrm{minimize}\;c\left(d\right)=U^{T}KU=\sum_{n=1}^{N}d_{n}^{p}u_{n}^{T}k_{n}u_{n}$$

(3) $$\;\mathrm{subject}\;\mathrm{to}\;V\left(d\right)=\kappa V_{0}$$

(4) KU=F

(5) $$0<d_{min}\leq d_{n}\leq 1$$

In Equations (2) and (4), U and F are the global displacement and force vectors, K is the global stiffness matrix, $u_{n}$ and $k_{n}$ are the element displacement vector and stiffness matrix, respectively, and *N* is the number of elements. Simultaneously, a global volume constraint can be applied for a specified volume fraction κ, where $V_{0}$ in Equation (3) stands for the design domain volume. The constraints are imposed on the design variables, and $d_{min}$ in Equation (5) is a nonzero minimum relative density introduced in order to avoid singularity. The design process consists of the redistribution of materials and parts that are not necessary from an objective point of view and are selectively removed.

In order to solve the above formulated problem, numerous algorithms based on various concepts have been proposed. Among them, the optimality criterion [30,32,33], the level-set method [34,35], the proportional topology optimization [36], the cellular automata approach [22,23], the implementation of a special function based on sorted compliances [31] and select other approaches, e.g., References [37,38], may serve as examples.

## 3. The Hybrid Algorithm

In this paper, the concept of a hybrid algorithm for a minimum compliance topology optimization is proposed. The main idea that stands behind it is to take two different approaches and to build a new one which takes advantage of the capabilities of both of them. The hybridization is successfull if the efficiency of the performance of the new algorithm finally outperforms the component ones running separately.

### 3.1. Concept

As to the optimization procedure, the sequential approach has been adapted, meaning that, for each iteration, the structural analysis performed for the optimized element is followed by the local updating process. For the iterative process, each subsequent step is realized based on the results of the former one. Changing the preceding configuration may influence the result of the current iteration step. For example, in a minimization process, it is possible that the modified result of the preceding iteration can result in finding, in the current iteration, a solution that is closer to the optimum, as compared to the one obtained without such a modification.

In a practical realization, for a single algorithm, the actual configuration is always updated based on the results obtained in the previous iteration, so that a potential modification would have to be enforced somehow. In the case of two algorithms, at each iteration step, there are two resulting configurations available. From these, only one is selected as the input for the next iteration. This means that, for each algorithm, its resulting configuration can be replaced by a configuration found by the second one. In what follows, the implementation of the same input configuration of the design variables into two algorithms results in different, possibly better, solutions. This can be a background for creating a hybrid algorithm.

While selecting algorithms for a hybridization it is important to search among those of different natures. For example, a combination of a gradient-based algorithm, which is more exploitive, and a heuristic one, which is more explorative, might be a good idea. The trade-off between exploitation and exploration may cause obtained solutions to be better than the ones resulting from a separate implementation of the algorithms. It is worth stressing that, in the hybridization concept, the clue is the possible exchange of design variables layouts between both algorithms at each iteration step.

For the purpose of the present paper, the well-recognized algorithm presented by Sigmund [30] as Algorithm (1) and the one quite recently proposed by Bochenek and Mazur [31] as Algorithm (2) have been selected. It is worth underlining that the process of topology generation for both algorithms is based on the same scheme. The difference is only in the form of the local update rule. In the case of the updating procedure described in Reference [30], the new values of design variables in iteration (t+1) are calculated based on the design variables in current iteration (t) according to the rule specified by

(6) $$d_{n}^{\left(t+1\right)}=d_{n}^{\left(t\right)}B_{n}^{\eta }$$

The quantity $B_{n}$ in Equation (6) is proportional to the sensitivity of the compliance function

(7) $$\frac{\partial c}{\partial d_{n}}=-pd_{n}^{\left(p-1\right)}u_{n}^{T}k_{n}u_{n}$$

while the numerical damping coefficient η equals 0.5. According to Reference [30], the mesh-independency filter can be additionally applied, thus modifying the element sensitivities. The modified element sensitivity is then calculated as a weighted sum of the sensitivities obtained for the elements forming a specified neighborhood around this element.

The idea of the heuristic concept of Algorithm (2) discussed in Reference [31] is as follows. Based on the results of the structural analysis, the values of local compliances are evaluated for *N* elements/design elements. Next, the compliances are sorted in ascending order, and the subsets of elements of the smallest and of the largest compliance values are selected. In what follows, $N_{1}$ and $N_{2}$ are specified, and f(n)=−1 if $n<N_{1}$ and f(n)=1 if $n>N_{2}$. For design elements of intermediate compliances, $N_{1}\leq n\leq N_{2}$ values of a specially adapted monotonically increasing function are assigned. In this case, the linear function of *n* has been adapted:

(8) $$f\left(n\right)=\frac{2n-N_{2}+N_{1}}{N_{2}-N_{1}}.$$

The local update rule applied to a design element $d_{n}$ is now constructed based on the values of the function f(n) defined in Equation (8) evaluated for this element and for *M* neighboring elements forming a user-specified neighborhood:

(9) $$d_{n}^{\left(t+1\right)}=d_{n}^{\left(t\right)}+\frac{1}{M+1}\left[\sum_{k=1}^{M}f(k)+f\left(n\right)\right]m.$$

The quantity *m* in Equation (9) stands for an admissible change of design variable value. It is worth underlining that the same neighborhood as for the Algorithm (1) has been selected here.

### 3.2. Performance

**Figure 1:**
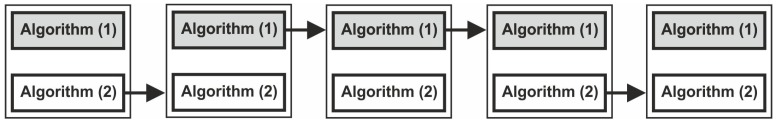
The hybrid algorithm performance. Switching between the update rules depending on the current compliance values resulting from an application of both approaches.

The numerical algorithm has been built in order to implement the above proposed concept. In a numerical implementation of the hybrid algorithm, the design variables are updated at each iteration step using both approaches. and the better one from the obtained solutions, in terms of the objective function value, is selected for the next iteration, as shown in Figure 1.

Simultaneously, a global volume constraint can be applied for a specified volume fraction. The volume constraint is implemented in each iteration when local update rules have been applied to all elements. In practice, the design variable multiplier is introduced, and then, its value is sought for so as to fulfill the volume constraint. As a result, the generated topologies preserve a specified volume fraction of a solid material during the optimization process.

**Figure 2:**
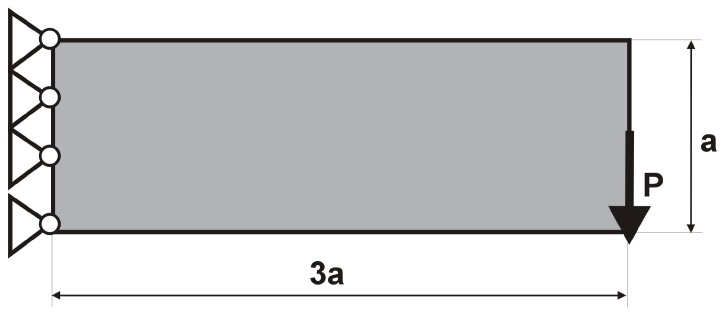
The design domain, loads and supports.

**Figure 3:**
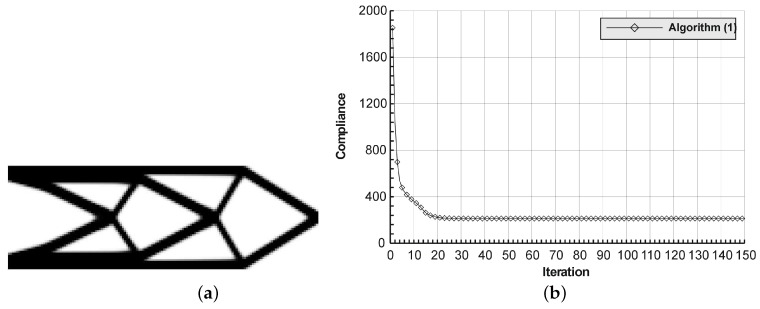
The generation of an optimal topology using Algorithm (1). (**a**) The final topology and (**b**) the compliance history.

**Figure 4:**
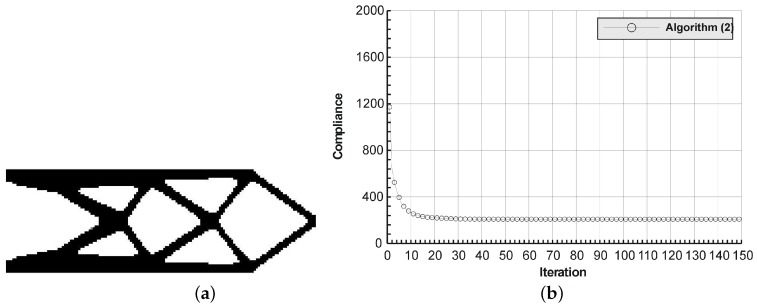
The generation of an optimal topology using Algorithm (2). (**a**) The final topology and (**b**) the compliance history.

**Figure 5:**
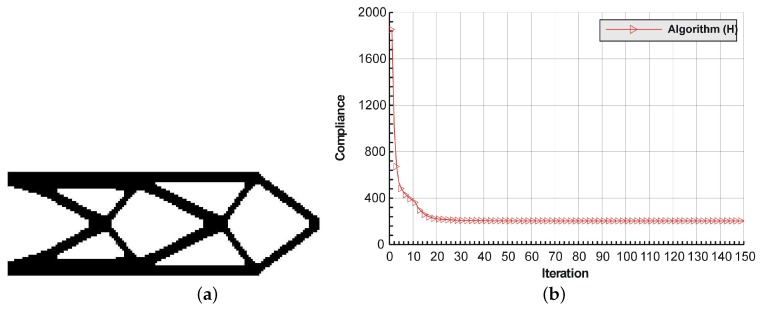
The generation of an optimal topology using Algorithm (H). (**a**) The final topology and (**b**) the compliance history.

**Figure 6:**
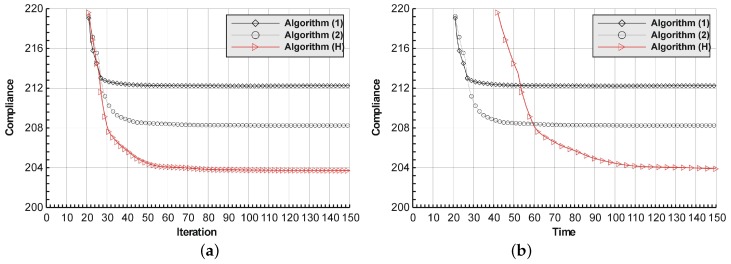
A comparison of the performance of the components Algorithms (1) and (2) running separately and the hybrid one: the iteration control (**a**) and the time control (**b**).

The detailed calculations are performed now for the introductory example in order to show how the hybrid algorithm works. The rectangular cantilever structure shown in Figure 2 has been chosen. The structure consists of 7500 elements, i.e., 150 × 50 square elements in a mesh discretization. For load P = 100 N, a = 50 mm, material data: E = 10 GPa, ν = 0.3 and volume fraction 0.4, minimal compliance topologies have been generated. The final topology together with the iteration history obtained with Algorithm (1) are shown in Figure 3. The compliance value for the final topology equals 212.24 Nmm. The results of the generation of minimal compliance topology using Algorithm (2) are given in Figure 4. This time, the compliance value for the final topology equals 206.94 Nmm. Finally, the implementation of the hybrid algorithm leads to the results presented in Figure 5. In this case, the compliance value for the final topology equals 203.70 Nmm. The fast convergence to the optimal solution is observed. The iteration histories for all above runs have been gathered in Figure 6, where a comparison of the performance of the component algorithms running separately and the hybrid one has been highlighted. One can observe that the hybrid algorithm allowed for the topology of the lowest objective function value to be obtained.

The additional analysis has been performed. Computations have been repeated several times, starting from different randomized initial configurations. The averaged values of the resulting compliances are 212.23 Nmm for Algorithm (1), 1208.82 for Algorithm (2) and 204.26 for Algorithm (H). The obtained values are close to the ones presented above, and the hybrid one still offers the lowest compliance value.

The duration of computations has been also discussed. The topology generator code has been written in Matlab, and a desktop computer Intel Core i7 CPU with 3.6 GHz processors and 8 GB RAM has been used. Based on 50 iteration runs, the approximate time spent for one iteration has been calculated for each. They were 4.3, 3.9 and 8.1 s for Algorithms (1), (2) and (H), respectively. As it can be observed, one iteration of the hybrid algorithm requires more computational time per iteration. This is caused by the additional structural analysis to be performed.

In order to illustrate this observation, the run time per iteration has been taken into account and Figure 6a has been slightly modified in the Figure 6b to show that the duration of one hybrid iteration is, in this case, about two times longer than that of the component algorithm. The unit of time in Figure 6b corresponds to approximately 4 s.

It is worth mentioning that the computational cost of conducting numerical calculations is generated mostly by the structural analysis. In this test, for a matter of comparison, the same structure analysis function as in Reference [30] has been implemented in all three tested algorithms. Since the attention has been focused on compliance values, the numerical code has not been optimized with respect to the run time.

**Figure 7:**
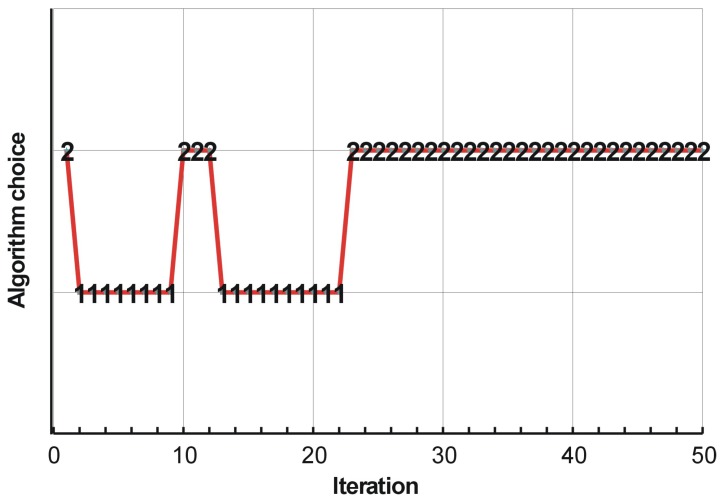
The hybrid algorithm performance. Switching between the update rules of Algorithms (1) and (2).

**Figure 8:**
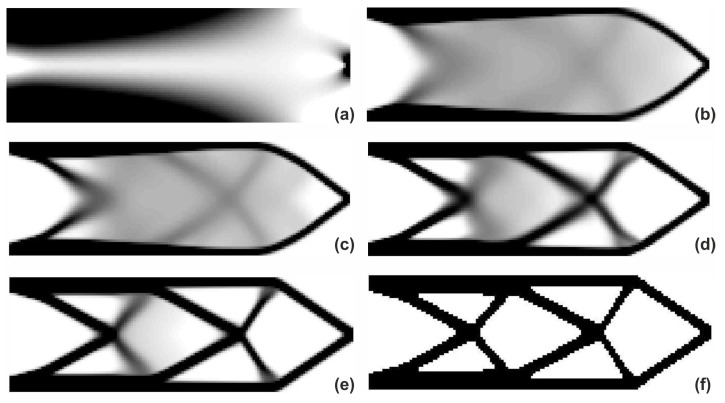
The overview of the topology generation process. The intermediate images for the selected iterations: 1 (**a**), 10 (**b**), 15 (**c**), 20 (**d**), 25 (**e**) and 50 (**f**).

The process of switching between update rules of Algorithms (1) and (2) while running the hybrid algorithm has been shown in Figure 7. In addition, the overview of the topology generation process has been presented in Figure 8.

## 4. Generation of Optimal Topologies

The discussion regarding the application of the hybrid algorithm into a generation of minimal compliance topologies continues in this section. A few illustrative test examples have been selected to show the algorithm performance.

**Figure 9:**
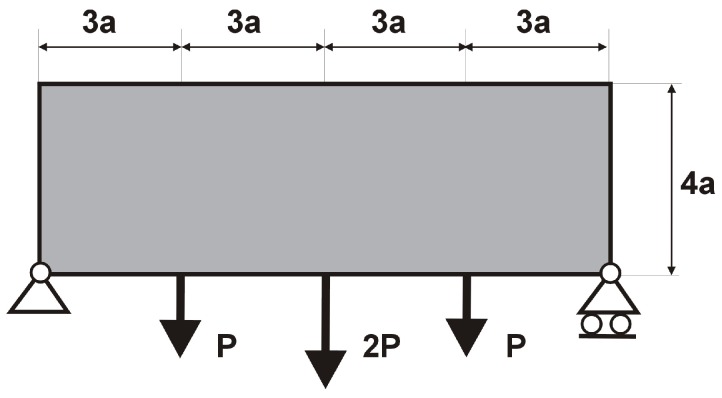
Test structure 1: The design domain, loads and supports.

**Figure 10:**
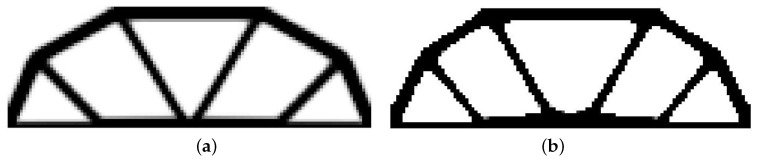
The generation of minimal compliance topologies for test structure 1. (**a**) The final topology using Algorithm (1). (**b**) The final topology using Algorithm (2).

**Figure 11:**
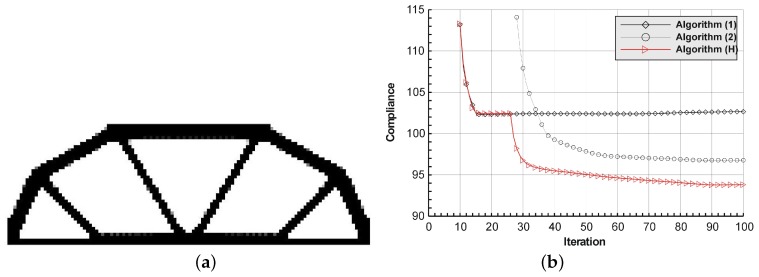
The performance of the hybrid algorithm applied to test structure 1. (**a**) The final topology using hybrid Algorithm (H). (**b**) A comparison of the performance of the component Algorithms (1) and (2) running separately and the hybrid one.

**Figure 12:**
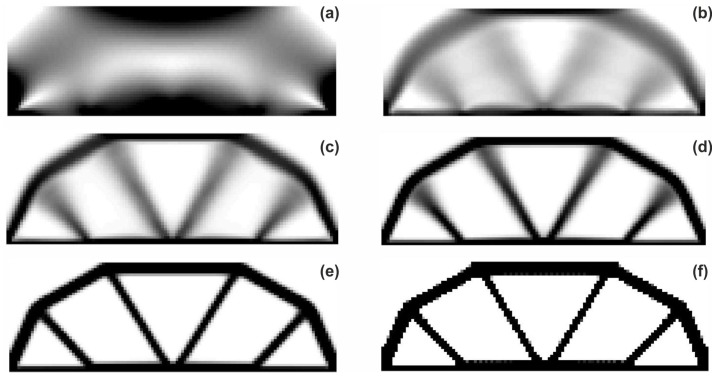
The overview of the topology generation process. The intermediate images for the selected iterations: 1 (**a**), 4 (**b**), 6 (**c**), 8 (**d**), 20 (**e**) and 100 (**f**).

The rectangular beam structure shown in Figure 9 has been chosen as test example 1. The structure consists of 4800 elements, i.e., 120 × 40 square elements in a mesh discretization. For load P = 50 N, a = 10 mm, material data: E = 10 GPa, ν = 0.3 and volume fraction 0.3, minimal compliance topologies have been generated. The final topologies obtained with Algorithm (1) and Algorithm (2) are shown in Figure 10. The compliance values for the final topologies equal 102.68 Nmm and 96.69 Nmm, respectively. The implementation of the hybrid algorithm leads to the result presented in Figure 11a. In this case, the compliance value for the final topology equals 93.71 Nmm. The run time per iteration were 1, 1 and 2 s for Algorithms (1), (2) and (H), respectively. In addition, the iteration histories for all algorithm runs have been gathered in Figure 11b, where a comparison of the performances of the component algorithms running separately and the hybrid one have been highlighted. In Figure 12, the selected intermediate topologies together with the final one for Algorithm (H) have been presented.

**Figure 13:**
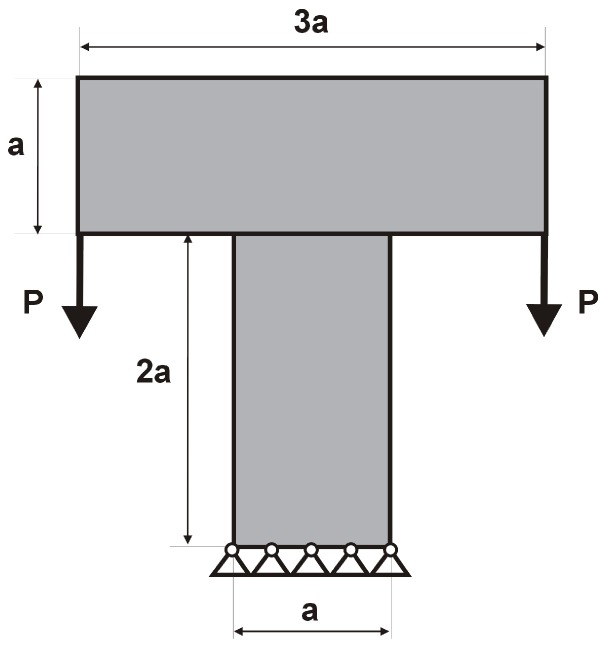
Test structure 2: The design domain, loads and supports.

**Figure 14:**
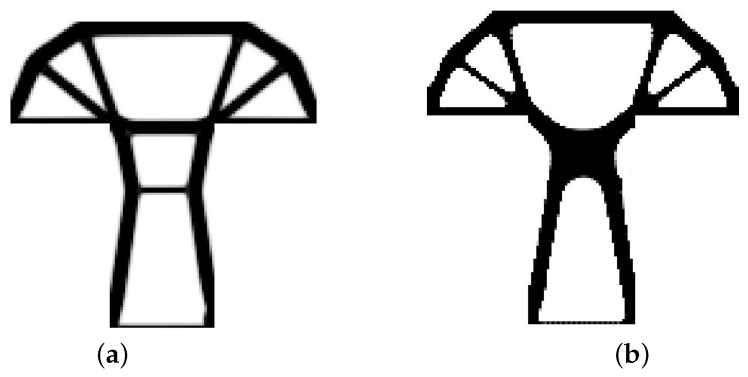
The generation of minimal compliance topologies for test structure 2. (**a**) The final topology using Algorithm (1). (**b**) The final topology using Algorithm (2).

**Figure 15:**
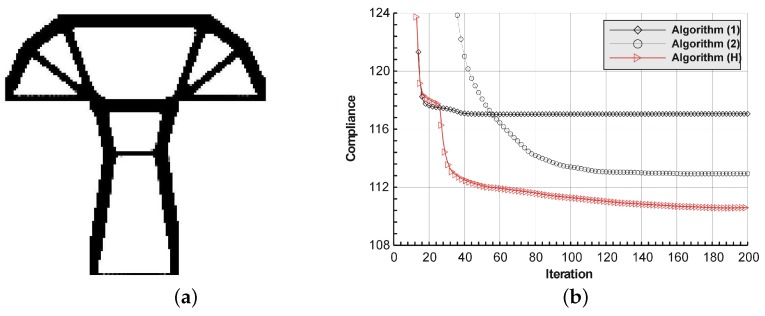
The performance of the hybrid algorithm applied to test structure 2. (**a**) The final topology using hybrid Algorithm (H). (**b**) A comparison of the performance of the component Algorithms (1) and (2) running separately and the hybrid one.

**Figure 16:**
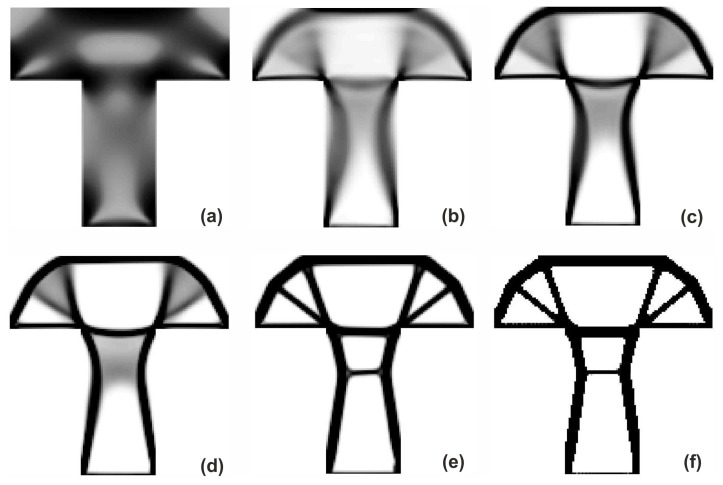
The overview of the topology generation process. The intermediate images for the selected iterations: 1 (**a**), 4 (**b**), 6 (**c**), 8 (**d**), 20 (**e**) and 100 (**f**).

The T-structure shown in Figure 13 has been introduced as test example 2. The structure consists of 14,400 elements in a mesh discretization. For load P = 100 N, a = 60 mm, material data: E = 10 GPa, ν = 0.3 and volume fraction 0.2, minimal compliance topologies have been generated. In Figure 14, the final topologies obtained with Algorithm (1) and Algorithm (2) are shown. The compliance values for the final topologies equal 117.04 Nmm and 112.93 Nmm, respectively. The implementation of the hybrid algorithm leads to the result presented in Figure 15a for which the compliance value is equal to 110.58 Nmm. The run times per iteration were 17, 17.3 and 34.1 seconds for Algorithms (1), (2) and (H), respectively. In Figure 15b, the iteration histories for all algorithms have been gathered and a comparison of the performance of the component algorithms running separately and the hybrid one have been highlighted. An overview of the topology generation process has been presented in Figure 16.

**Figure 17:**
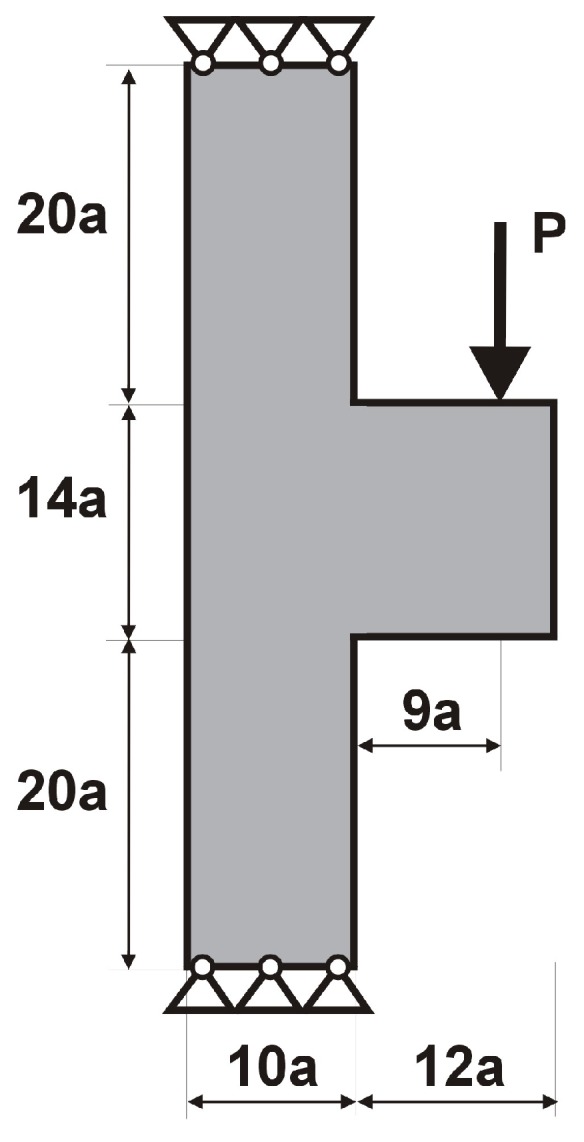
Test structure 3: The design domain, loads and supports.

**Figure 18:**
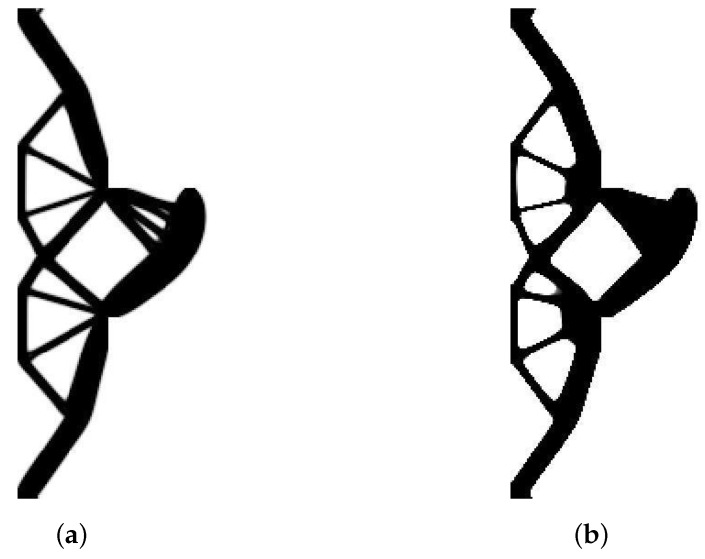
The generation of minimal compliance topologies for test structure 3. (**a**) The final topology using Algorithm (1). (**b**) The final topology using Algorithm (2).

**Figure 19:**
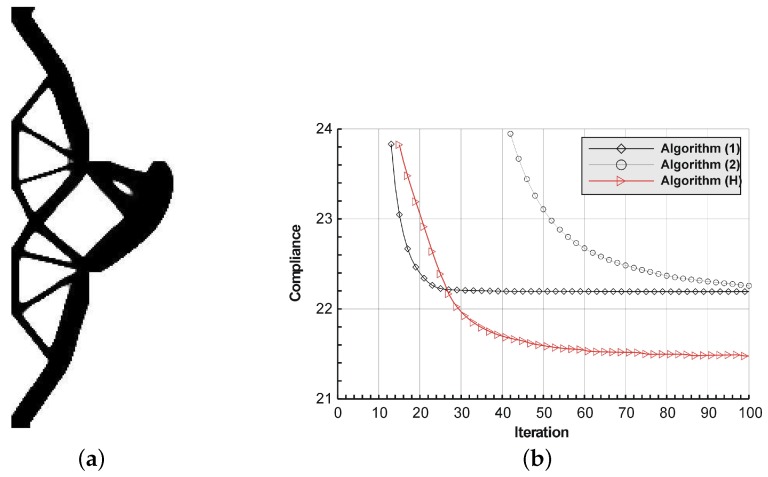
The performance of the hybrid algorithm applied to test structure 3. (**a**) The final topology using hybrid Algorithm (H). (**b**) A comparison of the performance of the component Algorithms (1) and (2) running separately and the hybrid one.

**Figure 20:**
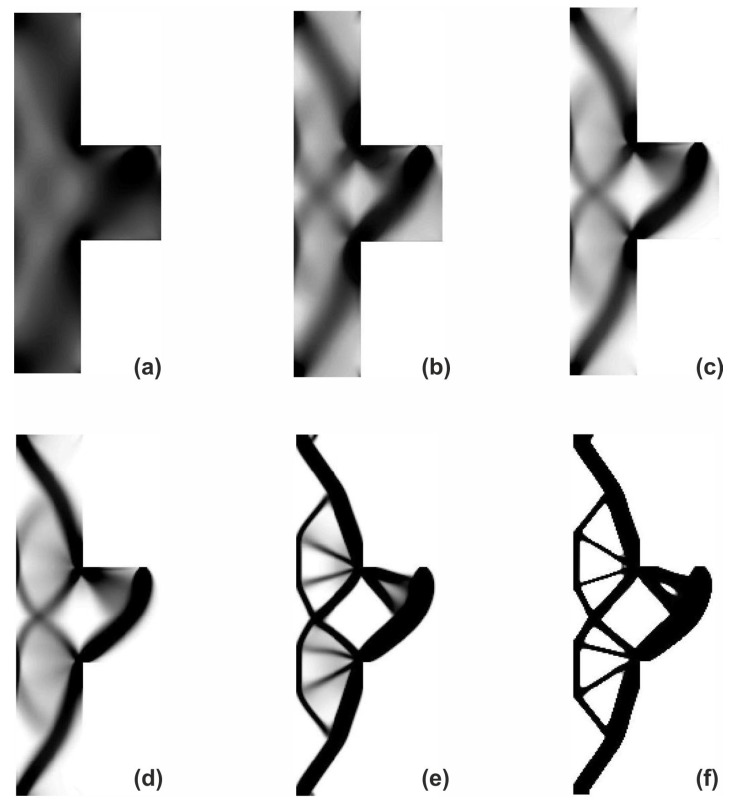
The overview of the topology generation process. The intermediate images for the selected iterations: 2 (**a**), 4 (**b**), 6 (**c**), 8 (**d**), 20 (**e**) and 100 (**f**), respectively.

The structure shown in Figure 17 has been chosen as test example 3. The structure consists of 17,700 elements in a mesh discretization. For load P = 100 N, a = 5 mm, material data: E = 10 GPa, ν = 0.3 and volume fraction 0.25, minimal compliance topologies have been generated. The final topologies obtained with Algorithm (1) and Algorithm (2) are shown in Figure 18. The compliance values for the final topologies equal 22.19 Nmm and 22.26 Nmm, respectively. The implementation of the hybrid algorithm leads to the result presented in Figure 19a. In this case, the compliance value for the final topology equals 21.47 Nmm. The run times per iteration were 79, 85.2 and 166 s for Algorithms (1), (2) and (H), respectively. In addition, the iteration histories for all algorithms have been gathered in Figure 19b, where a comparison of the performance of the component algorithms running separately and the hybrid one have been highlighted. In Figure 20, the selected intermediate topologies together with the final one have been presented.

**Figure 21:**
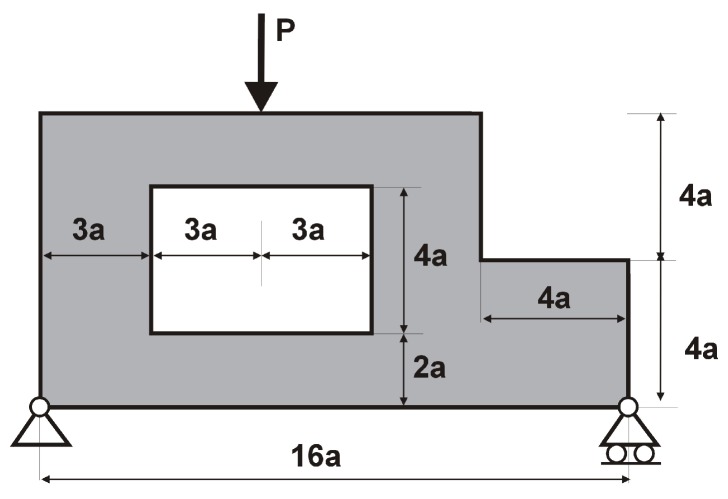
Test structure 4: The design domain, loads and supports.

**Figure 22:**
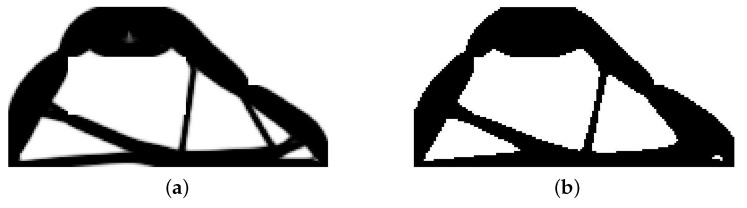
The generation of minimal compliance topologies for test structure 4. (**a**) The final topology using Algorithm (1). (**b**) The final topology using Algorithm (2).

**Figure 23:**
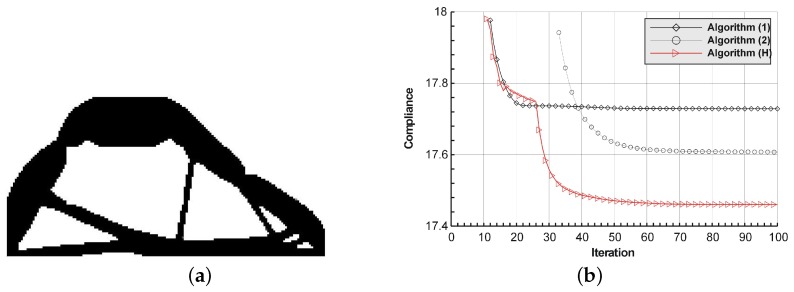
The performance of the hybrid algorithm applied to test structure 4. (**a**) The final topology using hybrid Algorithm (H). (**b**) A comparison of the performance of the component Algorithms (1) and (2) running separately and the hybrid one.

**Figure 24:**
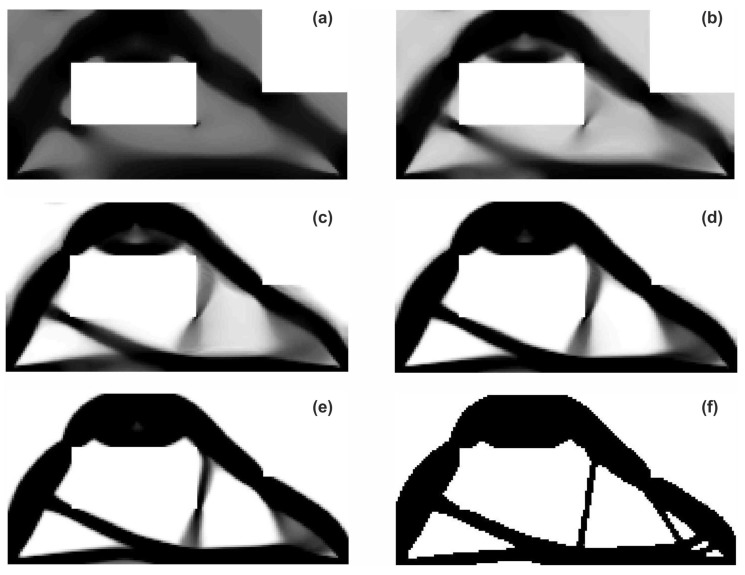
The overview of the topology generation process. The intermediate images for the selected iterations: 2 (**a**), 4 (**b**), 6 (**c**), 8 (**d**), 10 (**e**) and 100 (**f**).

The structure shown in Figure 21 has been proposed as test example 4. The structure consists of 8800 elements in a mesh discretization. For load P = 100 N, a = 10 mm, material data: E = 10 GPa, ν = 0.3 and volume fraction 0.4, minimal compliance topologies have been generated. The application of Algorithm (1) and Algorithm (2) result in finding the final topologies shown in Figure 22. The compliance values for the final topologies equal 17.73 Nmm and 17.61 Nmm, respectively. The implementation of the hybrid algorithm leads to the result presented in Figure 23a for which the compliance value is equal to 17.46 Nmm. The run times per iteration were 12.3, 12.8 and 25 s for Algorithms (1), (2) and (H), respectively. In Figure 23b, the iteration histories for all algorithms have been gathered and a comparison of the performance of the component algorithms running separately and the hybrid one have been highlighted. An overview of the topology generation process has been presented in Figure 24.

Although the aim of the paper is to present the original concept of a hybrid topology generator, which can work better than the base algorithms running separately, it is also worth making a comparison of the results obtained here with ones that can be found for the considered structures using other existing approaches. The algorithm based on the optimality criterion Top88 [32], the one implementing the level-set method Levelset88 [34] and the one utilizing a proportional topology optimization concept PTOc [36] have been chosen. The results have been gathered in Table 1. It can be seen that the hybrid algorithm proposed in this paper allows the results to be found, which can be better in terms of the objective function values as the ones obtained with the use of other approaches selected for the comparison purposes.

## 5. The Engineering Example

**Figure 25:**
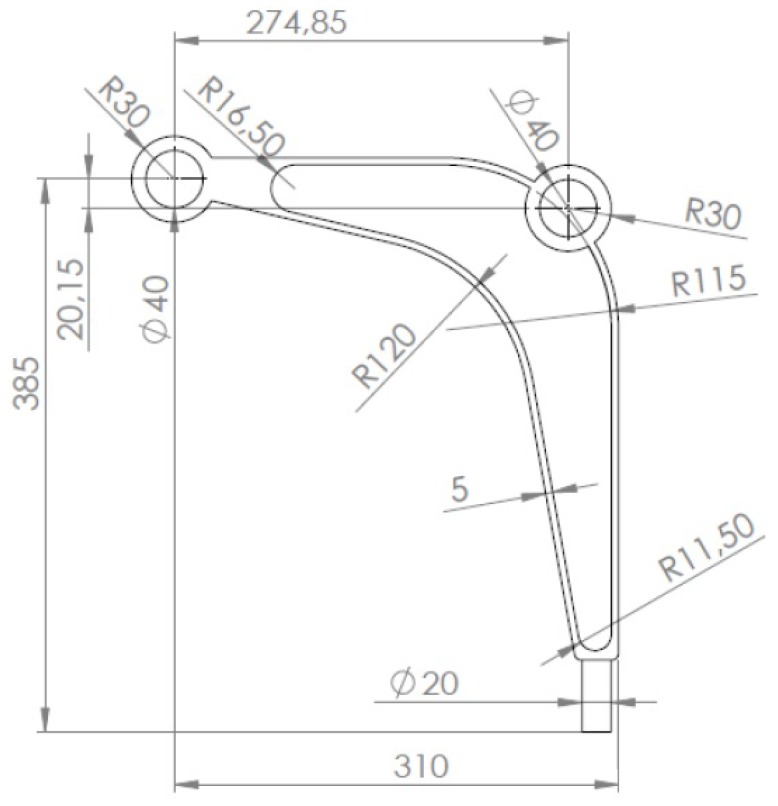
The control arm dimensions.

**Figure 26:**
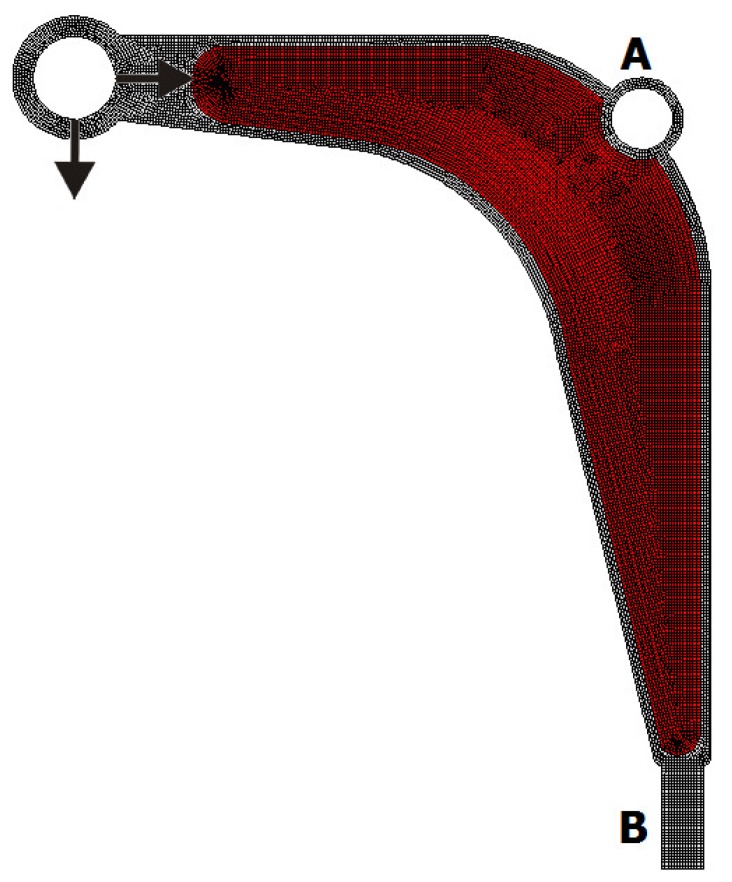
The control arm: The design domain, loads and supports.

Following the test examples of the previous section, an engineering example of a structural topology generation using the introduced approach is presented. The model of a control arm structure presented in Figure 25 has been chosen for this purpose. The Young modulus of the employed material equals 210 GPa with a Poisson ratio of 0.28. The structure consists of a non-optimized region presented in Figure 26 as a grey area and a design domain presented as a red area. The structure is discretised using 16 304 finite elements. The volume fraction is assumed to be equal 0.4. The structure is loaded by two concentrated forces: a horizontal force equal to 7000 N and a vertical force equal to 2700 N. The horizontal displacement of nodes in the inner bound of the round hole A are equal zero, while all nodes in area B are fixed.

**Figure 27:**
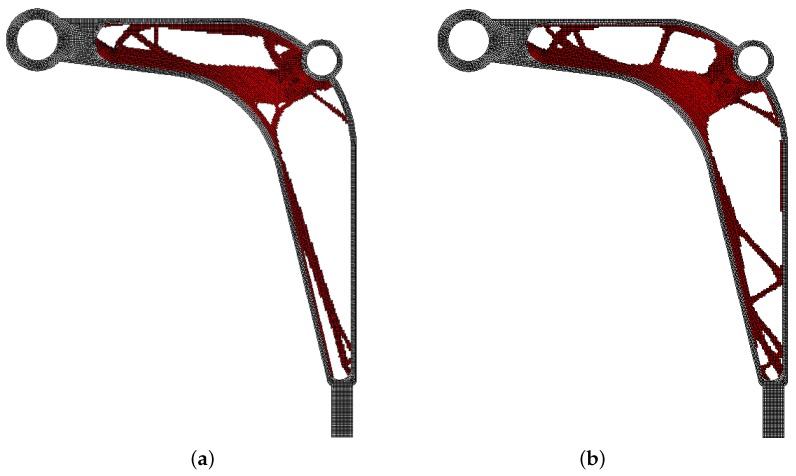
The generation of minimal compliance topologies for the control arm structure. (**a**) The final topology using Algorithm (1). (**b**) The final topology using Algorithm (2).

**Figure 28:**
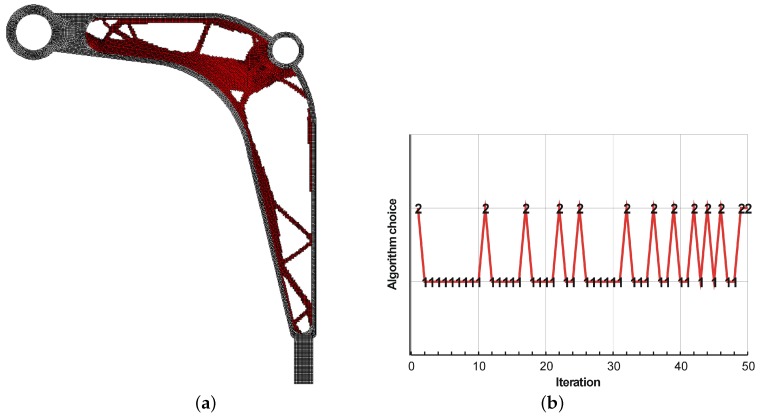
The final topology using hybrid Algorithm (H) (**a**). The hybrid algorithm performance: Switching between the update rules of Algorithms (1) and (2) (**b**).

The final topologies obtained with Algorithm (1) and Algorithm (2) are shown in Figure 27. The compliance values for the final topologies equal 12,372.02 Nmm and 12,001.16 Nmm, respectively. The implementation of the hybrid algorithm leads to the result presented in Figure 28a. In this case, the compliance value for the final topology equals 11,919.15 Nmm. The run times per iteration were 10.8, 10.8 and 18 seconds for Algorithms (1), (2) and (H), respectively. The desktop computer AMD Phenom II X4 955 with 3.2 GHz processors and 8 GB RAM and with Ansys 12.1 as the finite element code were used. In addition, the process of switching between the update rules of Algorithms (1) and (2) have been shown in Figure 28b.

## 6. Concluding Remarks

The concept of the hybrid generator of topologies of a minimal compliance has been presented. The main idea that stands behind the present proposal is to take the advantage of the two selected component algorithms’ capabilities with a view to build an efficient topology generator performing better than the component ones running separately. In a numerical implementation of the hybrid algorithm, the design variables are updated at each iteration step using both approaches and the solution with a lower objective function value is selected for the next iteration. The numerical tests of the generation of minimal compliance structures have been performed for the selected structures. It has been confirmed that the proposed hybrid technique based on switching between the considered rules allows the final structures having lower values of compliance compared with the results of the application of basic algorithms running separately to be obtained.

The development of topology optimization algorithms is combined with their expected practical implementation to engineering optimization problems. It is worth mentioning that, for the performed numerical calculations, a computational cost is generated mostly by the structural analysis. To be specific, for the example discussed above, the time used for the structural analyses ranges from about 80 to 90 percent of the total run time. The optimization algorithm based on the proposed local update rules returns here an almost immediate response. In order to restrain the total time of optimization process, the users’ activity should be precisely focused on the implementation of efficient structural analysis tools. The step to meet these expectations has been done in this paper by implementing the profesional finite element code Ansys. This approach has been just presented in the prevous section discussing the engineering example.

As the supplement to the above discussion, the test example 3 (Figure 17) has been solved once again, this time using an engineering-oriented topology generator with an Ansys structural analysis included. The run times per iteration were 8.4, 10.8 and 19 s for Algorithms/update rules (1), (2) and (H), respectively. One can observe that the run time per iteration can be reduced about 10 times as compared with the Matlab test algorithms. It has to be stressed that restraining the computing duration was possible due to an implementation of the efficient structural analysis system—Ansys.

Although in the present paper, the two algorithms have been hybridized, it is worth underlining that, from a software/algorithm point of view, it is possible to involve more update rules for a comparison. This, of course, does not mean that in all cases, the solution can be improved. Moreover, implementing hybridization must increase the duration of the topology generation process because of extra structural analyses. All in all, this issue requires further research and software development. It is expected that this must be a trade-off between broadening the set of available structure layouts and the duration of computations. The potential increase in number of algorithms involved may require, in our opinion, the implementation of parallel computing techniques into a topology generator. The open question for further investigations remains also the choice of component algorithms to create the efficient hybrid one: some of the remarks that have been already discussed in this paper. Nevertheless, it is worth pointing out that, when implementing the described technique, it is possible that one of the hybridized algorithms will outperform the other, meaning that the better solution in terms of objective function will always be a result of the same update rule. One can call this a neutral performance. Only one algorithm is active. The solution is not better than that obtained by the algorithms running separately, but it is not worse either.

Summing up the presentation and discussion included in this paper, one can state that the gate to the hybrid optimization of structural topologies has been just opened slightly.

## Figures and Tables

**Table 1:** A comparison of the results. The values of compliance (Nmm) obtained using the algorithms considered in the paper and select other ones are presented. The same number of iterations has been performed for all tests.

Algorithm	Test Structure 1	Test Structure 2	Test Structure 3	Test Structure 4
Algorithm (1) [30]	102.68	117.04	22.19	17.73
Algorithm (2) [31]	96.69	112.93	22.26	17.61
Hybrid algorithm	93.71	110.58	21.47	17.46
Top88 [32]	102.18	112.80	22.33	17.72
Levelset88 [34]	101.62	111.33	21.44	17.69
PTOc [36]	102.60	117.41	22.47	17.98
